# Arabidopsis Glutaredoxin S17 Contributes to Vegetative Growth, Mineral Accumulation, and Redox Balance during Iron Deficiency

**DOI:** 10.3389/fpls.2017.01045

**Published:** 2017-06-19

**Authors:** Han Yu, Jian Yang, Yafei Shi, Jimmonique Donelson, Sean M. Thompson, Stuart Sprague, Tony Roshan, Da-Li Wang, Jianzhong Liu, Sunghun Park, Paul A. Nakata, Erin L. Connolly, Kendal D. Hirschi, Michael A. Grusak, Ninghui Cheng

**Affiliations:** ^1^USDA/ARS Children’s Nutrition Research Center, Department of Pediatrics, Baylor College of Medicine, HoustonTX, United States; ^2^College of Chemistry and Life Science, Zhejiang Normal UniversityJinhua, China; ^3^Department of Horticultural Sciences, Texas A&M University, College StationTX, United States; ^4^Department of Horticulture, Forestry and Recreation Resources, Kansas State University, ManhattanKS, United States; ^5^Department of Plant Science, Penn State University, University ParkPA, United States; ^6^Vegetable and Fruit Improvement Center, Texas A&M University, College StationTX, United States; ^7^USDA/ARS Red River Valley Agricultural Research Center, FargoND, United States

**Keywords:** iron deficiency, oxidative stress, redox homeostasis, glutaredoxin, Arabidopsis

## Abstract

Iron (Fe) is an essential mineral nutrient and a metal cofactor required for many proteins and enzymes involved in the processes of DNA synthesis, respiration, and photosynthesis. Iron limitation can have detrimental effects on plant growth and development. Such effects are mediated, at least in part, through the generation of reactive oxygen species (ROS). Thus, plants have evolved a complex regulatory network to respond to conditions of iron limitations. However, the mechanisms that couple iron deficiency and oxidative stress responses are not fully understood. Here, we report the discovery that an *Arabidopsis thaliana* monothiol glutaredoxin S17 (AtGRXS17) plays a critical role in the plants ability to respond to iron deficiency stress and maintain redox homeostasis. In a yeast expression assay, AtGRXS17 was able to suppress the iron accumulation in yeast ScGrx3/ScGrx4 mutant cells. Genetic analysis indicated that plants with reduced *AtGRXS17* expression were hypersensitive to iron deficiency and showed increased iron concentrations in mature seeds. Disruption of *AtGRXS17* caused plant sensitivity to exogenous oxidants and increased ROS production under iron deficiency. Addition of reduced glutathione rescued the growth and alleviates the sensitivity of *atgrxs17* mutants to iron deficiency. These findings suggest AtGRXS17 helps integrate redox homeostasis and iron deficiency responses.

## Introduction

Iron is an essential mineral nutrient for plants (Guerinot and Yi, 1994; Briat et al., 2015). It serves as a metal cofactor required for hundreds of metabolic enzymes in the energy-yielding electron transfer reactions of respiration and photosynthesis (Briat et al., 2007; Balk and Schaedler, 2014). Perturbations in iron homeostasis can lead to cytotoxicity in the plant cell, reduction of growth and organ development, and eventually chlorosis and reduced crop yield (Connolly and Guerinot, 2002; Briat et al., 2007). Therefore, iron sensing and uptake from the soil, translocation within the plant, and intracellular storage and trafficking are tightly regulated in plants (Curie and Briat, 2003; Hell and Stephan, 2003; Jeong and Guerinot, 2009; Conte and Walker, 2011; Kobayashi and Nishizawa, 2012).

The iron deficiency response is thought to be controlled by a complex regulatory network involving multiple signaling pathways and its interplay with hormones (Hindt and Guerinot, 2012; Xia et al., 2015). Early studies indicate that adaptation to iron deficiency requires remodeling of the photosynthetic apparatus to minimize the photooxidative damage caused by reactive oxygen species (ROS) (Moseley et al., 2002). Genome-wide analyses of both transcript and protein expression profiles reveal significant changes in the expression of genes and/or proteins involved in antioxidant and oxidative stress response pathways (O’Rourke et al., 2007; Forner-Giner et al., 2010; Urzica et al., 2012; Zamboni et al., 2012; Lopez-Millan et al., 2013). In particular, expression of genes encoding glutaredoxins (Grxs) and thioredoxins (Trxs) is significantly enhanced under iron deficiency (Urzica et al., 2012). Furthermore, hydrogen peroxide (H_2_O_2_) production is increased in roots of plants grown under iron deficiency (Le et al., 2016). Nonetheless, the role of ROS and the function of redox-regulatory proteins in iron deficiency response regulation has not been well defined.

Grx and Trx enzyme systems help to control cellular redox potential in plants (Meyer et al., 2009). Grxs are ubiquitous small heat-stable disulfide oxidoreductases conserved in both prokaryotes and eukaryotes (Lillig et al., 2008) and are important in redox regulation and stress response (Sanchez-Riego et al., 2013; Considine and Foyer, 2014). There is growing evidence that plant Grxs have diverse functions in transcriptional regulation of defense responses and flower development (Xing et al., 2005; Ndamukong et al., 2007; Werner and Schmülling, 2009; La Camera et al., 2011), antioxidative stress (Cheng et al., 2006; Cheng, 2008; Laporte et al., 2012), redox signaling (Zaffagnini et al., 2012), hormonal regulation and environmental adaptation (Sundaram and Rathinasabapathi, 2010; Cheng et al., 2011). Monothiol Grxs are first identified in yeast (ScGrx3, -4, and -5) and bacteria (Grx4) that have a single cysteine residue in the putative active motif (Rodriguez-Manzaneque et al., 1999; Fernandes et al., 2005). This group of Grxs is conserved across species and accumulating evidence suggests they play a unique function in regulating iron homeostasis (Herrero and de la Torre-Ruiz, 2007; Lillig et al., 2008; Rouhier et al., 2010; Stroher and Millar, 2012). Yeast ScGrx5 encodes a mitochondrial monothiol Grx, which is required for biogenesis of iron-sulfur clusters (Rodriguez-Manzaneque et al., 2002; Uzarska et al., 2013), whereas ScGrx3 and ScGrx4, through interactions with iron-regulatory transcription factors, like Aft1 and Aft2, and coactivators like Fra/BolA proteins, globally modulate iron uptake, intracellular sensing, and trafficking in yeast cells (Ojeda et al., 2006; Pujol-Carrion et al., 2006; Kumanovics et al., 2008; Mercier and Labbé, 2009; Muhlenhoff et al., 2010; Li et al., 2011; Li and Outten, 2012; Vachon et al., 2012; Tamayo et al., 2016). In plants, Arabidopsis and Poplar monothiol Grxs, such as AtGRXS14 (AtGRXcp), AtGRXS15 (AtGRX4), AtGRXS16 and AtGRXS17, bind a Fe-S cluster and are able to complement yeast ScGrx5 function in Fe-S cluster assembly when expressed in yeast mutant cells (Cheng et al., 2006; Bandyopadhyay et al., 2008; Cheng, 2008; Li et al., 2010; Liu et al., 2013; Knuesting et al., 2015). However, the function of plant monothiol Grxs in iron regulation and stress responses *in planta* remains to be explored.

Arabidopsis AtGRXS17 is one of four “CGFS” type monothiol Grxs in Arabidopsis (Lemaire, 2004) with one Trx-like domain at its N-terminal region and three “CGFS” containing Grx domains at its C-terminus (Cheng et al., 2006; Herrero and de la Torre-Ruiz, 2007). Our previous studies indicate that AtGRXS17 is essential for post-embryonic growth and hormonal responses in plants under elevated temperature (Cheng et al., 2011). Meanwhile, ectopic expression of AtGRXS17 enhances stress tolerance (Wu et al., 2012; Hu et al., 2015). AtGRXS17 interacts with plant BolA proteins in an *in vitro* study (Couturier et al., 2014) and AtGRXS17 appears to be able to bind an iron-sulfur (Fe-S) cluster (Knuesting et al., 2015), suggesting that AtGRXS17 plays an important role in iron homeostasis. In the present report, we utilize yeast expression studies and reverse genetics in Arabidopsis to study the function of AtGRXS17 under iron deficiency stress. Our findings demonstrate that AtGRXS17 plays an important role in protecting plants from iron deficiency induced oxidative damage.

## Materials and Methods

### Reagents

All chemicals were purchased from Sigma–Aldrich (St. Louis, MO, United States) unless stated otherwise. Murashige and Skoog (MS) medium was purchased from Caisson Laboratories Inc (North Logan, UT, United States). AtGRXS17 polyclonal antibody was made in-house using the full-length Arabidopsis AtGRXS17 recombinant protein. This antibody does not cross react with other AtGRXs. Rabbit polyclonal antiserum against rubisco large subunit (form I and II) was purchased from Agrisera (Agrisera AB, Sweden).

### Plasmid DNA, Yeast Transformation, and Iron Content Assay

Yeast strains, expression plasmids, and the transformation protocol were described previously (Wu et al., 2012) (See detailed description in Supplementary Materials). Yeast cells were grown in nutrient-enriched medium (YPD) overnight, harvested, washed twice with distilled water, then dried for metal ion measurement as previously described (Cheng et al., 2006).

### Plant Materials and Growth Conditions

Wild type (ecotype Columbia, Col-0), *atgrxs17* KO, and *AtGRXS17* RNAi lines were described previously (Cheng et al., 2011) (See detailed description in Supplementary Materials). For growth assays, wild type and mutant seeds were surface-sterilized, germinated, and grown on one-half strength MS medium (plus 0.5% sucrose), which consists of 50 μM Fe, solidified with 0.8% agar or ½ MS medium supplemented with various concentrations of H_2_O_2_. Iron sufficient and deficient medium were made following the previous report with minor modification (Connolly et al., 2002; Barberon et al., 2014). In brief, 1 L of synthetic medium (SM) was made containing 0.47 g Ca(NO_3_)_2_.4H_2_O, 0.1307 g K_2_SO_4_, 0.1602 g MgSO_4_.7H_2_O, 0.0136 g KH_2_PO_4_, 0.5 g MES, 1 mL of 1000× micronutrients (0.01 mM H_3_BO_3_, 0.1 μM MnSO_4_, 0.05 μM CuSO_4_, 0.05 μM ZnSO_4_, and 5 nM Na_2_MO_4_). The pH was adjusted to 6 with 1 M NaOH. To make iron-sufficient medium, 50 μM (final concentration) FeEDTA was added to the SM plus 0.5% sucrose. For iron-deficient medium, 300 μM (final concentration) Ferrozine was added into the SM plus 0.5% sucrose as well. For iron stress assays, wild type, *atgrxs17* KO, and *AtGRXS17* RNAi seeds were germinated and grown on ½ MS medium for 5 days, then transferred and grown on iron sufficient or deficient medium for 6 days before measuring primary root length of seedlings or for 11 days before fresh weight of seedlings was measured. For iron deficiency stress rescue experiments, 250 μM (final concentration) GSH was added into the iron sufficient or iron deficient medium.

### Plant Mineral Ion Concentration Measurement

Wild type, *atgrxs17* KO, and *AtGRXS17* RNAi seeds were germinated and grown in soil (Sunshine Mix, Sun Gro Horticulture, Agawam, MA, United States) in a controlled greenhouse or growth chamber at 22°C. Mature leaves were collected from 5-week-old plants, while seeds were harvested from mature plants. Four independent experiments were done for each treatment of each genotype. Elemental analysis was performed using inductively coupled plasma–optical emission spectroscopy as described previously (Farnham et al., 2011).

### Ferric Chelate Reductase Assay

Ferric chelate reductase assays were performed as previously described (Grusak et al., 1990). In brief, wild type, *atgrxs17* KO, and *AtGRXS17* RNAi seeds were germinated and grown on ½ MS medium for 10 days at 22°C under 16 h light and 8 h dark. Seedlings were transferred and grown on iron sufficient or deficient medium for 3 additional days, then rinsed twice with distilled water and the entire root system of each seedling was submerged in 1 mL of assay solution [100 μM Fe(III)-EDTA and 300 μM Na_2_-BDPS] for 1hr at room temperature in the dark. The amount of Fe(II)-BPDS_3_ was measured by reading the absorbance at 562 nM. The Fe(III)-reductase activity was calculated as μmol Fe(II) per gram root fresh weight per hour. Six samples were measured for each genotype and three independent experiments were conducted.

### RNA Isolation, cDNA Synthesis, and qRT-PCR Analysis

Wild-type seeds were germinated and grown on ½ MS medium for 10 days at 22°C under 16 h light and 8 h night. Seedlings were transferred and grown on iron sufficient or deficient medium for growth of 6 or 24 h. Twenty seedlings for each treatment were pooled for RNA isolation. Three independent experiments were conducted. Total RNA was extracted from wild-type seedlings and purified RNA samples underwent reverse transcription to yield cDNA. qRT-PCR was performed using the SYBR Green-based system on the Bio-Rad CFX96^TM^. Primers were used for *AtGRXS17*: tgctgtgccttatttcgtcttc (forward) and tctgcaccctcaagtgtatcca (reverse); and for *ACTIN1*serving as the internal control: gtgctcgactctggagatggtgtg (forward) and cggcgattccagggaacattgtgg (reverse).

### Western Blot Analysis

Wild type, *atgrxs17* KO, and *AtGRXS17* RNAi seeds were germinated and grown on ½ MS medium for 14 days and then transferred onto iron deficient or sufficient medium for additional 3 days before being harvested. Seedling tissue homogenates (20 μg per lane) were run on SDS–PAGE gel and western blot analysis was conducted following an established procedure (Liu et al., 2013) (See detailed description in Supplementary Materials). Rabbit antiserum against AtGRXS17 was used at dilution of 1:500 and Anti-RbcL antibody was used at 1:2500 dilution.

### ROS Production Measurement

Wild type, *atgrxs17* KO, and *AtGRXS17* RNAi seeds were germinated and grown on ½ MS medium for 5 days, then transferred and grown on iron sufficient or deficient medium for 3 days before seedlings were collected for measurement of ROS production in roots. Seedlings were transferred from agar plates into Eppendorf tubes containing 1 mL of cold PBS and were washed twice with 1 mL cold PBS. For ROS measurement, the roots were stained with 10 μM Dihydroethidium (DHE) (Camacho-Cristóbal et al., 2015) for 45 min to 1 h, washed once with PBS and left in PBS before imaging with a confocal microscope at 582 nm (excitation at 543 nm) for Texas Red. The mean fluorescence intensity (MFI) of root tips from six to ten randomly selected seedlings was quantified using ImageJ software.

## Results

### AtGRXS17 Is Able to Suppress the Yeast *grx3grx4* Iron Accumulation Phenotype

**FIGURE 1:**
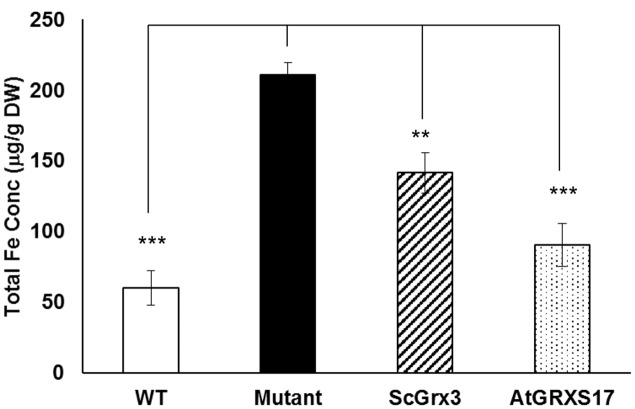
AtGRXS17 suppresses yeast *grx3grx4* iron accumulation phenotypes. Yeast wild type and *grx3grx4* cells expressing empty vector and plasmid DNA as indicated were grown in YPD medium overnight and whole cell iron concentrations were measured by inductively coupled plasma optical emission spectrometry. Results represent the mean value of three independent replications. Student’s *t*-test, ^∗∗^*p* < 0.01, ^∗∗∗^*p* < 0.001.

Arabidopsis AtGRXS17 suppresses the sensitivity of yeast *grx3grx4* cells to oxidative stress (Wu et al., 2012). In yeast, ScGrx3 and ScGrx4 play a critical role in iron uptake, trafficking, mitochondrial iron dynamics and homeostasis (Ojeda et al., 2006; Pujol-Carrion et al., 2006; Muhlenhoff et al., 2010). Disruption of both ScGrx3 and ScGrx4 results in the accumulation of iron in the cell (Pujol-Carrion et al., 2006) and expression of yeast ScGrx3 could rescue, at least in part, the accumulation of free iron in the *grx3grx4* mutant (**Figure 1**). When expressed in *grx3grx4*, AtGRXS17 was able to suppress the iron accumulation phenotype of *grx3grx4* cells (**Figure 1**). These results suggest that AtGRXS17 may function in iron regulation in plants.

### *AtGRXS17* Expression Is Induced by Iron Deficiency Stress

To understand the physiological function of AtGRXS17 in iron regulation in plants, the responsiveness of endogenous *AtGRXS17* to iron limiting conditions was examined by qRT-PCR. As shown in Supplementary Figure S1A, the level of *AtGRXS17* mRNA was increased about threefolds under iron deficiency conditions. *IRT1* was used as a control to indicate iron status as this gene induced under this condition (Connolly et al., 2002). AtGRXS17 protein levels were also increased in plants grown under iron deficiency compared to iron sufficient condition (Supplementary Figure S1B). The results suggest that AtGRXS17 may play an important role in response to iron deficiency in plants.

### *AtGRXS17* Loss-of-function Seedlings Are Sensitive to Iron Deficiency Stress

**FIGURE 2:**
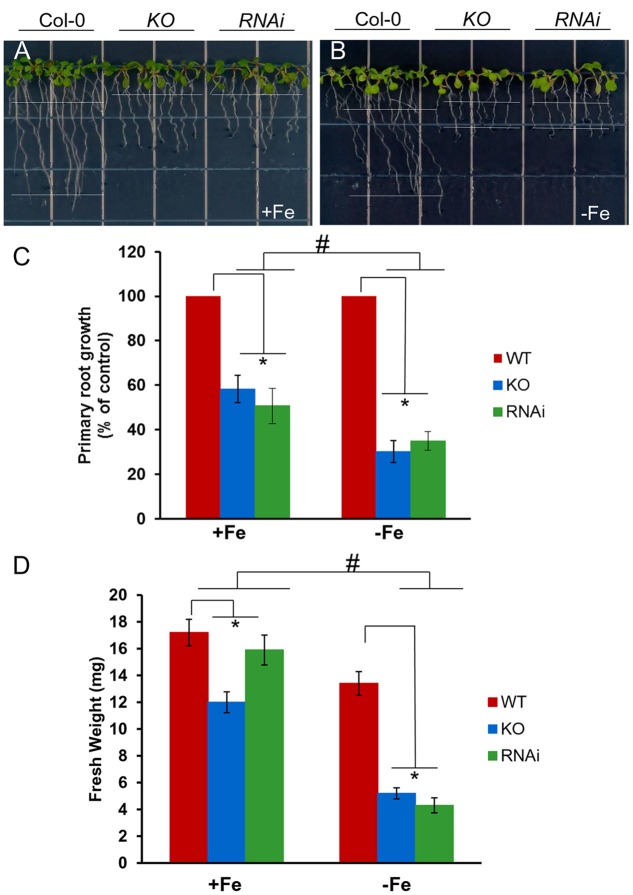
Loss of AtGRXS17 impairs seedlings growth under iron deficiency stress. **(A–C)**, Wild type control, *atgrxs17* KO, and *AtGRXS17* RNAi seeds were germinated and grown on ½ MS medium (0.5% Sucrose) for 5 days and then transferred onto Fe sufficient or deficient medium as indicated for 6 additional days of growth at 22°C. In **(C)**, primary root growth was measured and the growth rate was calculated relative to wild type controls. Statistical analysis using a two-way ANOVA. *n* ≥ 18. ^∗^*p* < 0.05, significance between WT controls and *atgrxs17* mutants; ^#^*p* < 0.05, significance between +Fe and –Fe treatments. **(D)** Wild type control, *atgrxs17* KO, and *AtGRXS17* RNAi seeds were germinated on ½ MS medium (0.5% Suc) for 5 days and then transferred onto iron sufficient or deficient medium as indicated for 11 days of growth at 22°C. Fresh weight was measured to compare mutants to wild type controls. Statistical analysis using a two-way ANOVA. *n* ≥ 12. ^∗^*p* < 0.05, significance between WT controls and *atgrxs17* mutants; ^#^*p* < 0.05, significance between +Fe and –Fe treatments.

To test the function of AtGRXS17 *in planta*, *atgrxs17* KO and *AtGRXS17* RNAi lines were generated (Supplementary Figure S1C). *AtGRXS17* loss-of-function seedlings (KO and RNAi lines) displayed strong growth inhibition of primary roots under iron deficiency (about 35% of wild-type root growth) compared to those grown on iron sufficient medium (about 60% of wild-type root growth) (**Figures 2A–C**). The overall growth of mutant seedlings as measured by fresh weight was decreased under iron deficiency compared to those grown on iron sufficient medium (**Figure 2D**). These findings indicate that AtGRXS17 helps to maintain plant growth under iron deficiency stress.

### *atgrxs17* Plants Accumulate More Iron in Seeds

**FIGURE 3:**
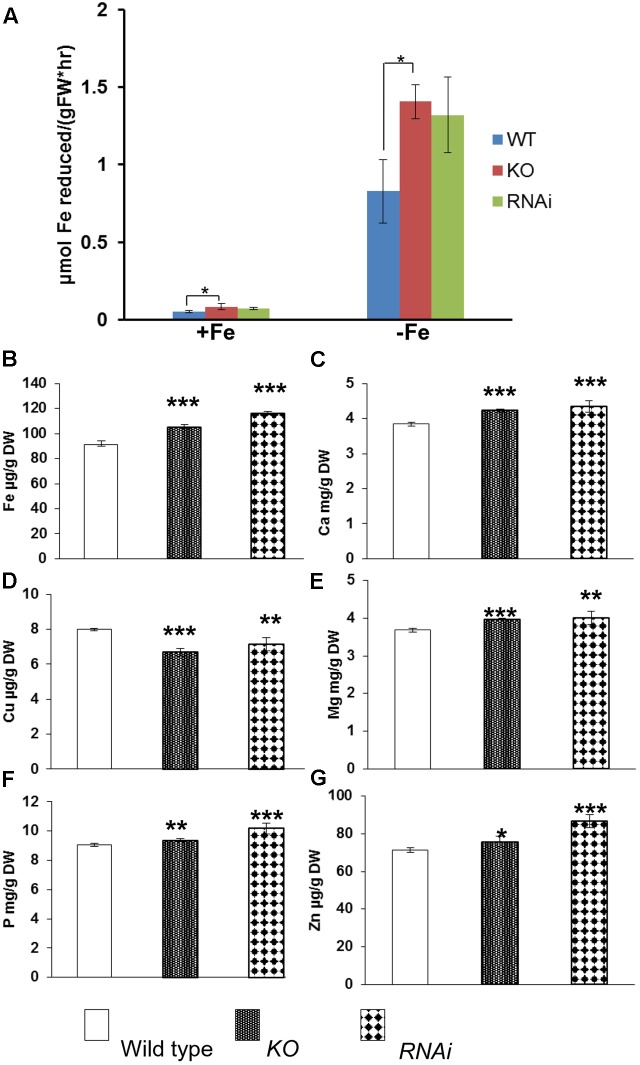
Iron reductase assay and metal ion concentrations in wild type, *atgrxs17* KO, and *AtGRXS17* RNAi seeds. **(A)**, Wild type, *atgrxs17* KO, and *AtGRXS17* RNAi seeds were germinated and grown on ½ MS medium for 10 days, then transferred to iron sufficient or deficient medium for 3 days. Root ferric reductase activity was measured. Student’s *t*-test, *n* = 6, ^∗^*p* < 0.05. **(B–G)**, Wild type, *atgrxs17* KO, and *AtGRXS17* RNAi seeds were germinated and grown in soil until mature. Seeds were harvested and dried. Whole seed Fe and other mineral concentrations were measured by inductively coupled plasma optical emission spectroscopy. Results represent the mean value of three independent replications. Student’s *t*-test, ^∗^*p* < 0.05; ^∗∗^*p* < 0.01; ^∗∗∗^*p* < 0.001.

To determine whether disruption of *AtGRXS17* affects ferric chelate reductase, both wild type control and mutant seedlings were grown on iron sufficient or deficient medium for 3 days. Root ferric chelate reductase activities of both mutant and wild-type seedlings were increased under iron deficiency compared to those under iron sufficient medium (**Figure 3A**). Compared to wild type controls, *atgrxs17* KO and *AtGRXS17* RNAi seedlings had higher reductase activities under both iron sufficient and deficient conditions, in which the increase of reductase activity in *atgrxs17* KO seedlings was significant (**Figure 3A**). When grown in soil under normal growth conditions, AtGRXS17 loss-of-function plants demonstrate subtle growth defects (Cheng et al., 2011; Knuesting et al., 2015). When mature leaves from 5-week-old mutant and wild type control plants grown under normal growth conditions were collected and iron concentrations were examined, total iron concentration in leaves of mutant plants was indistinguishable from wild type controls (data not shown). However, measurement of iron concentrations of dry seeds from mutant and wild-type plants indicated that AtGRXS17 loss-of-function plants demonstrated significantly higher iron concentrations in seeds compared to wild type controls (**Figure 3B**). Furthermore, mutant plants showed higher concentrations of other mineral ions, such as Ca, Mg, Zn, and P, while exhibiting decreased concentrations of Cu in seeds (**Figures 3C–G**).

### *atgrxs17* Seedlings Are Sensitive to Oxidative Stress and Increase ROS Production under Iron Deficiency Stress

**FIGURE 4:**
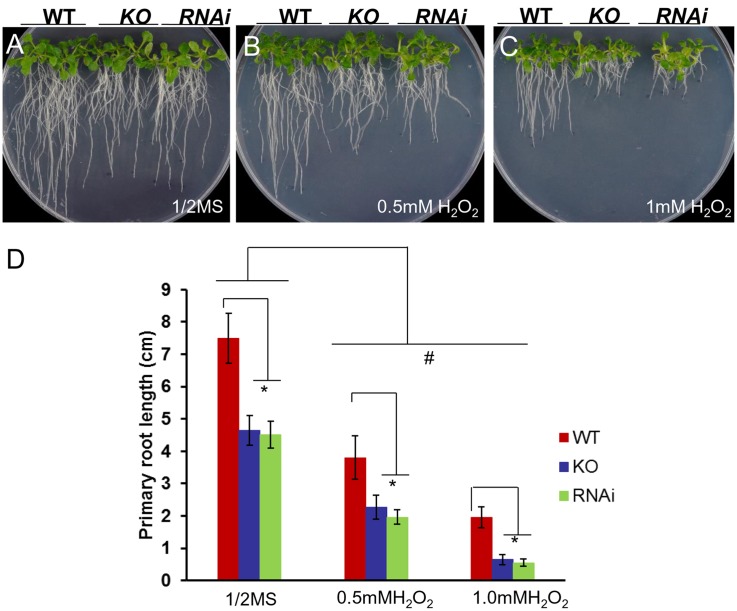
*atgrxs17* KO and *AtGRXS17* RNAi seedlings are sensitive to oxidative stress. Wild type, *atgrxs17* KO, and *AtGRXS17* RNAi seeds were germinated and vertically grown on ½ MS medium **(A)** and the same medium supplemented with 0.5 mM **(B)** and 1 mM **(C)** H_2_O_2_ for 10 days. **(D)** The length of primary roots was recorded. Statistical analysis using a two-way ANOVA. *n* ≥ 18. ^∗^*p* < 0.05, significance between WT controls and *atgrxs17* mutants; ^#^*p* < 0.05, significance between ½ MS medium and H_2_O_2_ treatments.

**FIGURE 5:**
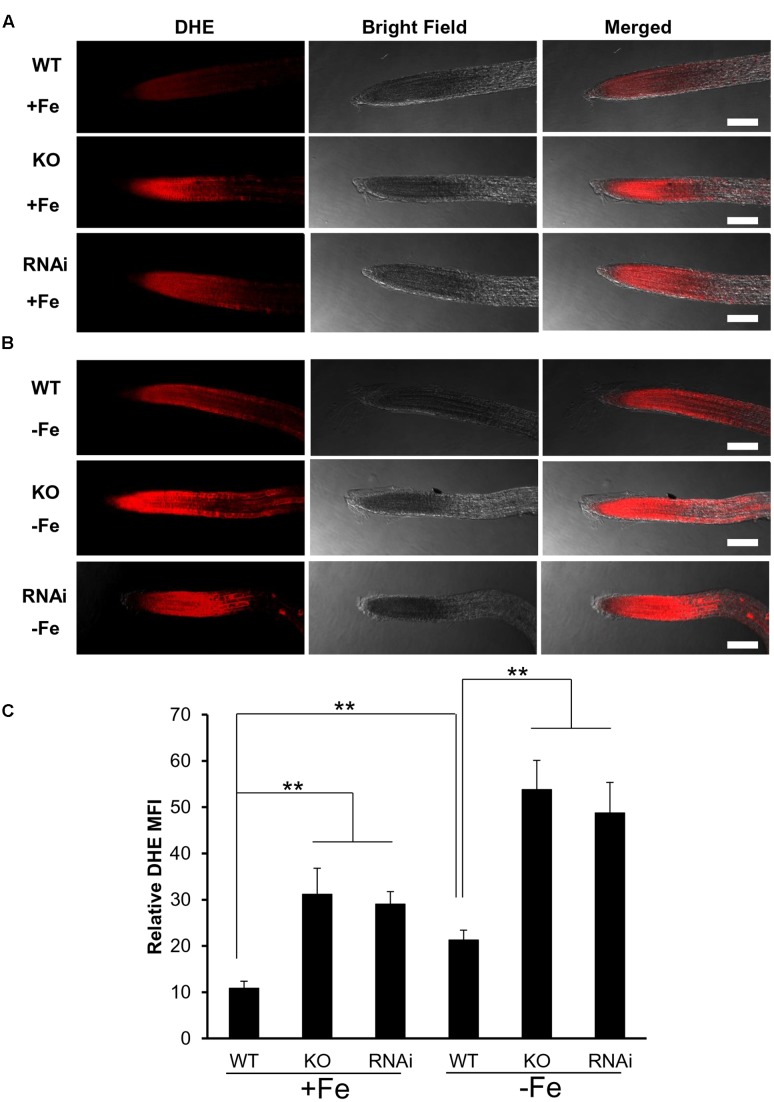
*atgrxs17* seedlings increase ROS production under iron deficiency stress. Wild type control, *atgrxs17* KO, and *AtGRXS17* RNAi seeds were germinated and vertically grown on ½ MS medium for 5 days, then transferred to and grown on iron sufficient or deficient medium for 3 days. Seedlings were stained with DHE to monitor ROS levels. Shown are representative images for iron sufficient **(A)** and deficient **(B)** treatments. Scale Bars = 50 μm. **(C)** The intensity of ROS signals were captured by confocal microscope and analyzed with Image J. Student’s *t*-test, *n* = 6–10, ^∗∗^*p* < 0.01.

When measuring H_2_O_2_ accumulation in *atgrxs17* KO seedlings by DAB staining, the root tips and the junction areas (between the hypocotyl and the root) display more intense staining than controls (Cheng et al., 2011). When grown on medium containing H_2_O_2_, the primary root growth of *atgrxs17* KO and *AtGRXS17* RNAi seedlings was significantly inhibited compared to wild type controls (**Figure 4**), suggesting that *AtGRXS17* loss-of-function plants are more sensitive to external oxidative stress. Previous studies have reported that iron deficiency induces gene expression response to oxidative stress in various species (O’Rourke et al., 2007; Zamboni et al., 2012) and a rapid increase in hydrogen peroxide (H_2_O_2_) production (Le et al., 2016). To ascertain whether iron deficiency stress induces ROS production and how AtGRXS17 affects this process, wild type control and mutant roots were stained with DHE, which enables the detection of ROS by fluorescence microscopy. Under iron sufficient condition, ROS production was increased in mutant root tips compared to wild type controls (**Figures 5A,C**), which is consistent with our previous report (Cheng et al., 2011). As expected, iron deficiency caused a significant increase of red fluorescence (ROS levels) in both wild type and mutant roots compared to that under iron sufficient condition (**Figures 5B,C**), in which enhancement of ROS production in *atgrxs17* KO and RNAi roots had expanded from the root tips to the elongation zone of the roots (**Figures 5A,B**). Thus, these findings indicate that AtGRXS17 plays a role in controlling oxidative stress induced by iron deficiency stress.

### Attenuation of *atgrxs17* Seedling Sensitivity to Iron Deficiency Stress by Reduced GSH

**FIGURE 6:**
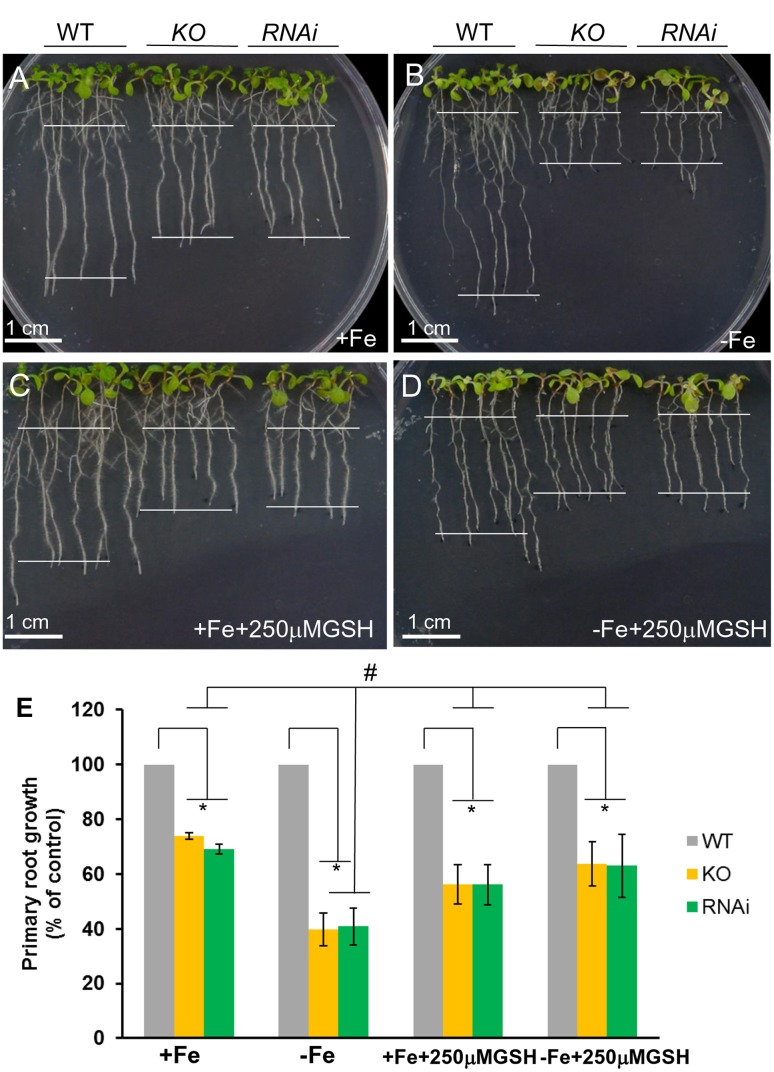
*atgrxs17* seedling sensitivities to iron deficiency stress are rescued by reduced glutathione (GSH). Wild type control, *atgrxs17* KO, and *AtGRXS17* RNAi seeds were germinated on ½ MS medium for 5 days and then transferred to and grown on Fe sufficient **(A)** or deficient **(B)** medium without or on Fe sufficient **(C)** or deficient **(D)** with addition of 250 μM reduced GSH as indicated for 6 days of growth at 22°C. In **(E)**, primary root length was measured and the growth rate was calculated relative to wild type controls. Statistical analysis using a two-way ANOVA. *n* ≥ 18. ^∗^*p* < 0.05, significance between WT controls and *atgrxs17* mutants; ^#^*p* < 0.05, significance between +Fe and –Fe treatments in the presence of reduced GSH.

To determine whether *AtGRXS17* loss-of-function seedling sensitivity to iron-deficiency stress was due to disruption of redox balance, *atgrxs17* KO and *AtGRXS17* RNAi seedlings were tested on iron sufficient and deficient conditions with addition of reduced GSH. When grown on iron sufficient and deficient conditions without addition of reduced GSH, the growth of primary roots of KO and RNAi seedlings was inhibited under iron deficient condition compared to that under iron sufficient condition (**Figures 6A,B,E**), while grown on iron sufficient and deficient conditions with addition of reduced GSH, the growth of primary roots of *atgrxs17* KO and *AtGRXS17* RNAi seedlings was indistinguishable under iron deficiency compared to that under iron sufficient condition (**Figures 6C–E**). These findings indicate AtGRXS17 modulates iron deficiency stress responses through mediation of redox homeostasis in plants.

## Discussion

Our genetic studies presented here offer insight into the relationship between redox regulation and iron homeostasis in plants. This work provides evidence that AtGRXS17 is involved in regulation of iron homeostasis in plants and helps to alleviate iron deficiency stress through mediating redox balance.

In yeast mutant cells, AtGRXS17 suppressed iron accumulation phenotypes (**Figure 1**). Interestingly, the suppression of iron accumulation in double mutant cells by AtGRXS17 appears to be stronger than ScGrx3 alone (**Figure 1**). This observation could be due to the fact that ScGrx3 and ScGrx4 functions are not completely overlapping. It has been shown that cytosolic and nuclear ScGrx3/ScGrx4 have distinct functions in iron regulation and homeostasis (Ojeda et al., 2006; Pujol-Carrion et al., 2006; Muhlenhoff et al., 2010). When ectopically expressed in yeast, AtGRXS17 is found both in the nucleus and the cytoplasm (Wu et al., 2012). It is possible that AtGRXS17 could rescue the yeast *grx3grx4* phenotype better due to its dual cellular localization in yeast cells.

In yeast, deletion of both ScGrx3 and ScGrx4 results in growth defects and enhanced sensitivity to oxidative stress caused by iron accumulation (Pujol-Carrion et al., 2006; Wu et al., 2012). Similarly, *AtGRXS17* loss-of-function plants displayed root and vegetative growth retardation under normal conditions (**Figures 2A,C,D**) (Cheng et al., 2011; Knuesting et al., 2015) and increased sensitivity to oxidative stress (**Figure 4**). This finding indicates that AtGRXS17 and its yeast orthologs have conserved functions. However, there is functional divergene between the plant and yeast genes as yeast *grx3grx4* cells use an iron chelator to alleviate oxidative stress (Pujol-Carrion et al., 2006). Meanwhile *AtGRXS17* loss-of-function seedlings were hypersensitive to iron deficiency (in the presence of iron chelator) (**Figure 2**).

Root iron reductase activity was significantly induced in wild type controls and mutants under iron deplete medium compared to that under iron sufficient medium (**Figure 3A**). It appears that *atgrxs17* KO and *AtGRXS17* RNAi seedlings has higher root iron reductase activities than wild type controls under both iron sufficient and deficient conditions (**Figure 3A**). The increase in root iron reductase activity, especially under iron sufficient medium, may not result in increased iron uptake and accumulation in mutant plants. In agreement with this, no difference in iron accumulation in mature leaves between *AtGRXS17* loss-of-function plants and wild type controls was observed when plants were grown in soil. However, iron concentration in mature seeds of mutant plants was slightly, but significantly increased compared to wild type controls (**Figure 3B**). This suggests that AtGRXS17 may modulate iron distribution within a plant. It is also possible that a reduction in seed yield of these mutant plants could have contributed to the elevated seed iron levels through a concentrating process (i.e., the same total partitioning of iron to seeds, but to a smaller pool of seeds). Higher concentrations of calcium, magnesium, phosphorus, and zinc were also seen in seeds of soil-grown mutant plants (**Figures 3C,E–G**). Unfortunately, seed yield was not measured in the current study. Previous research has shown that seeds comprise about 30% of whole-shoot iron content and about 15% of shoot mass at maturity in Arabidopsis (Waters and Grusak, 2008). Thus, even a moderate lowering of seed production in the mutants could explain a portion of the increased seed iron concentration in these plants. Previous studies have shown that AtGRXS17 loss-of-function plants display subtle growth defects (Cheng et al., 2011; Knuesting et al., 2015). Furthermore, when seedlings were grown on iron sufficient medium, the growth of *AtGRXS17* mutant primary roots was slower than that of wild type controls (**Figures 2A,C**, **6A,E**). Whether those growth defects are attributed to altered iron accumulation/distribution is not clear in the current study. We posit that the increased ROS production/oxidative stress (**Figures 5A,C**) are the causal factors for inhibition of root growth.

Iron deficiency causes cellular oxidative stress and induces antioxidant defense genes (pathways) including Grxs in plants and green algae (Thimm et al., 2001; Connolly and Guerinot, 2002; O’Rourke et al., 2007; Lan et al., 2011; Urzica et al., 2012; Lopez-Millan et al., 2013; Le et al., 2016). Reduced GSH contents are significantly decreased in Arabidopsis plants under iron deficiency, whereas ROS levels are drastically increased (Ramirez et al., 2013). Furthermore, addition of reduced GSH can alleviate the detrimental effects of iron deficiency through controlling ROS production/oxidative stress caused by iron deficiency (Ramirez et al., 2013). Our data revealed an increase of *AtGRXS17* expression (both mRNA and protein levels) in plants under iron deficiency (Supplementary Figure S1), while disruption of *AtGRXS17* significantly inhibited plant growth under the same condition (**Figure 2**). This finding demonstrates that AtGRXS17 is required for plant survival under iron deficiency stress. Our studies support the notion that AtGRXS17 may alleviate the iron deficiency stress through mediating redox balance. First, *atgrxs17* KO and *AtGRXS17* RNAi seedlings are sensitive to oxidative stress (**Figure 4**); Second, ROS production is significantly increased in mutant roots under iron deficiency (**Figures 5B,C**), which is a contributing factor to cause cell damage and impair plant growth. Third, although the intracellular GSH levels in both mutant seedlings and wild type controls were not measured in the current study, addition of reduced GSH is able to suppress, at least in part, the growth defects of mutant seedlings under iron deficiency (**Figure 6**). Furthermore, overexpression of *AtGRXS17* enhances antioxidant enzymatic activities in transgenic tomato plants (Wu et al., 2012). Taken together, these results indicate that AtGRXS17 is crucial for protecting plants from iron deficiency induced oxidative damage.

AtGRXS17, similar to ScGrx3/ScGrx4, is a Fe-S cluster binding protein (Knuesting et al., 2015) and is postulated to mediate iron or iron-sulfur cluster transfer processes (Philpott, 2012; Inigo et al., 2016). Disruption of both *ScGrx3* and *ScGrx4* in yeast drastically alters iron sensing, intracellular trafficking, and mitochondrial iron distribution through their bound iron-sulfur clusters (Muhlenhoff et al., 2010). Whether AtGRXS17 modulates iron deficiency responses through its bound cluster is yet to be determined. Interestingly, Arabidopsis BolA protein, an interacting partner of AtGRXS17, might play a role in iron metabolism and redox regulation independent of its iron-sulfur binding ability (Qin et al., 2015). We envision AtGRXS17 playing a myriad of roles *in planta*; however, studies directed at clarifying other AtGRXS17 functions require additional inquiry.

Recent advances have indicated that the interaction among multiple phytohormones, such as auxin, ethylene, and nitric oxide (NO), plays an important role in iron deficiency responses in plants (Schmidt et al., 2000; Seguela et al., 2008; Romera et al., 2011; Hindt and Guerinot, 2012). For example, recent reports indicate that auxin can regulate plant responses to iron deficiency through a NO-mediated signaling pathway (Chen et al., 2010; Jin et al., 2011). Furthermore, decreased auxin concentrations and polar auxin transport in auxin transporter mutants trigger up-regulation of iron deficient responsive genes (Xu et al., 2014). Our previous study demonstrates that AtGRXS17 is crucial for auxin response and function in temperature stress (Cheng et al., 2011). Whether AtGRXS17 mediates its effects on the iron deficiency response via modulation of auxin response pathways remains to be further investigated.

## Conclusion

AtGRX7 is part of the ensemble of plant genes that sense and respond to fluctuations in iron availability. Using heterologous expression and reverse genetic approaches, this work establishes that AtGRXS17 functions under iron limiting conditions to modulate plant growth, iron accumulation, and redox balance.

## Author Contributions

NC and MG designed the study and wrote the paper. HY, JY, JD, YS, ST, TR, D-LW, JL, SP, and NC performed and analyzed the experiments. PN, EC, and KH provided technical assistance and analysis and interpretation of data. All authors reviewed the results and approved the final version of the manuscript.

## Conflict of Interest Statement

The authors declare that the research was conducted in the absence of any commercial or financial relationships that could be construed as a potential conflict of interest.
