# Supplementary material for: Arabidopsis Glutaredoxin S17 Contributes to Vegetative Growth, Mineral Accumulation, and Redox Balance during Iron Deficiency
Source: Front Plant Sci. 2017 Jun 19;8:1045. doi: 10.3389/fpls.2017.01045 (PMC5474874; doi:10.3389/fpls.2017.01045)
Supplement: Supplementary file 1 [file Presentation_1.PDF]

Supplementary Figure S1

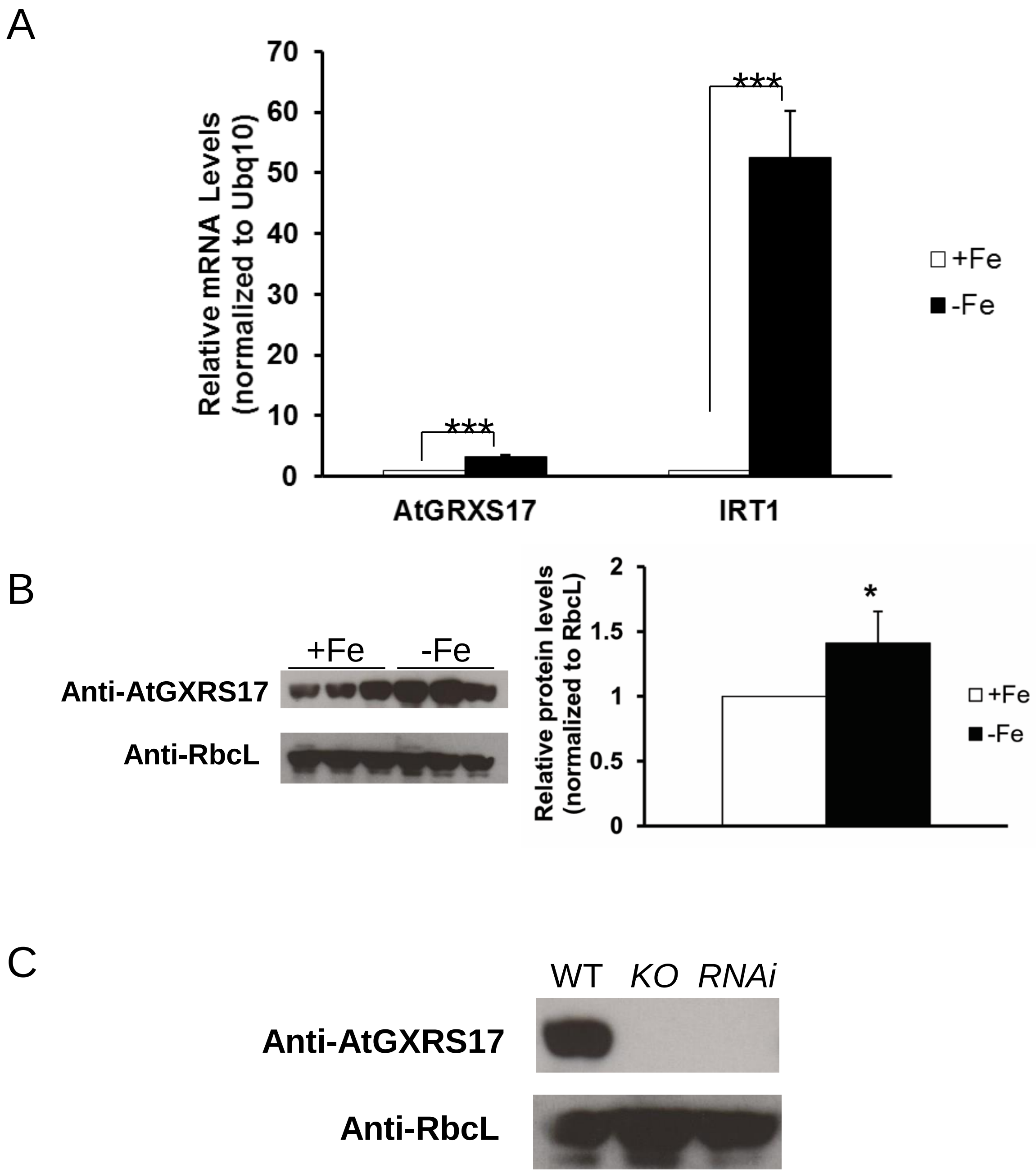

Figure S1 *AtGRXS17* gene expression under iron deficiency. Wild type seeds were germinated and grown on ½ MS medium for 14 days, then transferred to iron sufficient (+Fe) or deficient (-Fe) medium for 3 days. (**A**), Twenty seedlings from each treatment were collected and total RNA samples were extracted and made for cDNAs. q-PCR analysis was conducted with an *UBQ10* gene as an internal reference. *IRT1* gene used as a positive control for iron deficient treatment because it is known *IRT1* is highly induced under this condition. All results shown here are the means of three independent experiments. Student *t* test, ***$p< 0.001$. (**B**), Seedling tissue homogenates (20 µg per lane) were run on SDS-PAGE gel and Western blot analysis was conducted with rabbit antibodies against AtGRXS17 (1:500 dilution) and RbcL (1:2500 dilution), as loading controls. The densitometry intensity of western blots was calculated and quantified using Image J software. Student *t* test, n=3, *$p<0.05$. (**C**), Tissue homogenates (20 µg per lane) from wild type control, *atgrxs17* KO, and *RNAi* seedlings treated with iron deficiency stress were run western blot analysis with Anti-AtGRXS17 and Anti-RbcL antibodies.

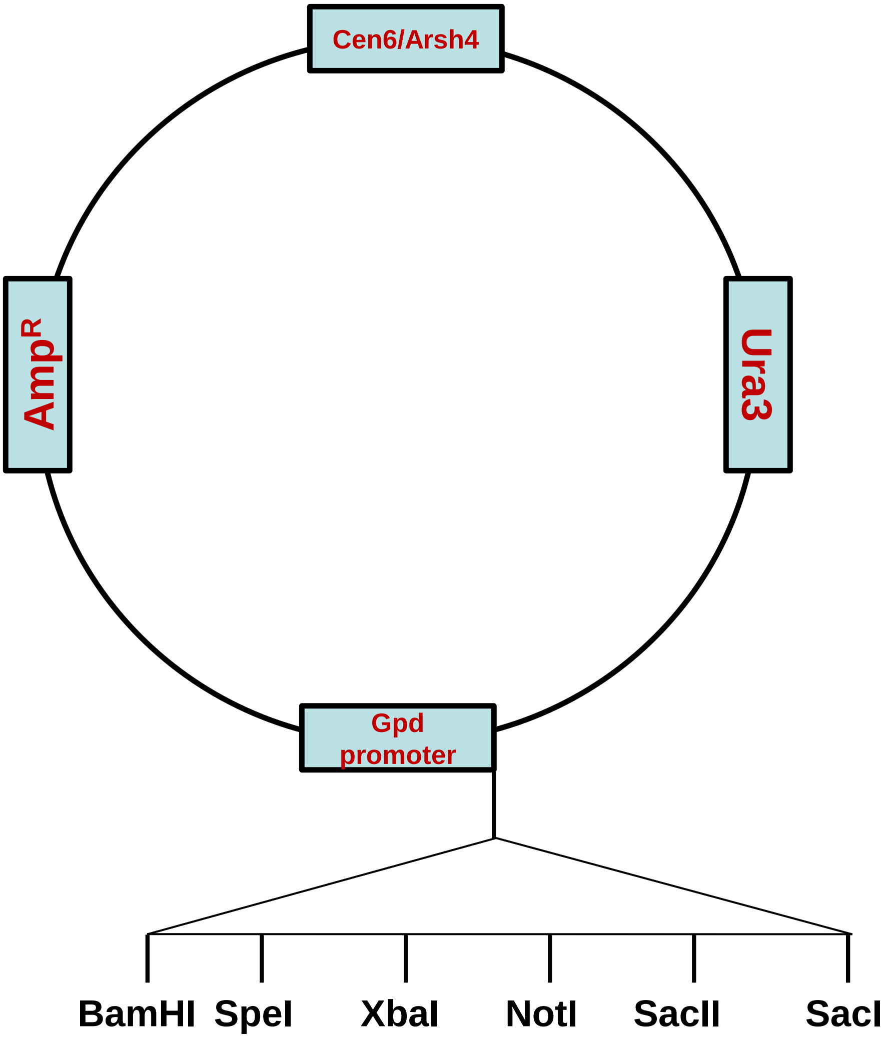

Figure S2 The plasmid map for AtGRXS17 yeast expression construct. The shuttle vector piUGpd consists of Ampicillin selection in bacteria and uracil selection in yeast. The Arabidopsis AtGRXS17 gene was cloned into piUGpd with BamHI/SacI site and driven by a Gpd promoter.

1. Yeast strains, expression plasmids, and transformation protocol

Saccharomyces cerevisiae wild-type strain CML235 (*MATα ura3-52 leu2Δ1 his3Δ200*) and *grx3grx4* double-mutant (*MATα ura3-52 leu2Δ1 his3Δ200 grx3::kanMX4 grx4::kanMX4*) were provided by Dr. Enrique Herrero (Universitat de Lleida, Lleida, Spain) (Rodriguez-Manzaneque et al., 1999) and used in all yeast experiments. To express AtGRXS17 in yeast cells, the full-length cDNA of AtGRXS17 was subcloned into yeast expression vector piUGpd (Supplementary Figure S2) (Nathan et al., 1999). Yeast cells were transformed using the LiOAc method (Gietz and Schiestl, 2007). In brief, yeast cells were grown in 10 mL of 2X YPD medium (1% Bacto yeast extract, 2% Bacto peptone, 80 mg/L of Adenine hemisulfate) with shaking overnight at 30°C. The cells were harvested by spinning at 3000g for 5 min and washed with 10 mL of sterile water twice. The following components were added in each transformation: 240 uL of PEG 3350 (50% w/v), 36 uL of 1M LiOAc, 50 uL of single-stranded carrier DNA (2.0 mg/mL), and 34 uL of plasmid DNA plus sterile water. The cells with the transformation components were resuspended and mixed by vortexing vigorously, and then placed in a water bath at 42°C for 40 min. after heat-shock, the cells were transferred to the microtube and centrifuged at 13,000g for 30s. The supernatant was discard and the cells were washed with sterile water twice, then resuspended in sterile water and plated on synthetic selection medium for grown at 30°C until the transformants were visible. The colonies were collected for subsequent experiments.

2. Isolation of AtGRXS17-null Alleles and Creation of AtGRXS17 RNAi Lines

An *atgrxs17* null allele and AtGRXS17 RNAi lines were isolated and generated in our previous study (Cheng et al., 2011). In brief, a T-DNA insertional mutant line, was obtained from the SALK T-DNA collection (SALK_021301). Homozygous plants from the T3 generation were obtained by PCR screening using an AtGRXS17 reverse primer (5’-TAGCTCGGATAGAGTTGCTTT-3’) and a T-DNA left border primer (5’-GCGTGG ACCGCTTGCTGCA-3’) for *atgrxs17* allele; an AtGRXS17 forward primer: 5’-ATG AGC GGT ACG GTG AAG GAT-3’ and the AtGRXS17 reverse primer were used for identifying the wild type. The location of the T-DNA insertion was determined by sequencing the PCR product. The *atgrxs17* allele was backcrossed to wild type to remove any potential unlinked mutations. To generate *AtGRXS17* RNAi lines, the AtGRXS17 full-length cDNA was cloned into the binary vector pCHF3 with opposite orientation. The antisense construct was transformed into Agrobacterium GV3101 strain, and then the positive strains were used to transform Arabidopsis Col-0 plants using the floral-dip method(Clough and Bent, 1998). The transgenic progeny were selected by kanamycin resistance. *AtGRXS17* expression levels in both *atgrxs17* knock-out and *AtGRXS17* RNAi plants were examined using semi-quantitative RT-PCR.

3. Western blot analysis

Wild type, *atgrxs17* KO, and *AtGRXS17* RNAi seedlings were treated under iron sufficient and deficient conditions for 3 days and harvested for tissue homogenates with the lysis buffer (50 mM HEPES pH 7.5, 150 mM NaCl, 10% Glycerol, 1% TritionX-100, 1 mM PMSF plus cocktail Inhibitor). Twenty micrograms of protein lysates were loaded and run on 12% SDS/PAGE gel and then transferred onto PVDF membrane. The blots were blocked in 5% non-fat milk in TBST for 1 hour at room temperature and then incubated with rabbit antiserum against AtGRXS17 at dilution of 1:500 or Anti-RbcL antibody at 1:2500 dilution for 3 hrs at room temperature. The blots were rinsed with PBST three times for 10 mins each and then incubated with goat anti-rabbit secondary

antibody for 30 min at room temperature. After that, the blots were rinsed with PBS-T three times for 10 mins each and then developed with Amersham ECL Select Western Blotting Detection Reagent. The densitometry intensity of western blots was calculated and quantified using Image J software.

References:

Cheng, N.H., Liu, J.Z., Liu, X., Wu, Q., Thompson, S.M., Lin, J., Chang, J., Whitham, S.A., Park, S., Cohen, J.D., Hirschi, K.D., 2011. Arabidopsis monothiol glutaredoxin, AtGRXS17, is critical for temperature-dependent postembryonic growth and development via modulating auxin response. J Biol Chem 286 (23), 20398-20406.

Clough, S.J., Bent, A.F., 1998. Floral dip: a simplified method for Agrobacterium-mediated transformation of Arabidopsis thaliana. Plant J 16 (6), 735-743.

Gietz, R.D., Schiestl, R.H., 2007. High-efficiency yeast transformation using the LiAc/SS carrier DNA/PEG method. Nature protocols 2(1), 31-34.

Nathan, D.F., Vos, M.H., Lindquist, S., 1999. Identification of SSF1, CNS1, and HCH1 as multicopy suppressors of a Saccharomyces cerevisiae Hsp90 loss-of-function mutation. Proc Natl Acad Sci U S A 96
(4), 1409-1414.

Rodriguez-Manzaneque, M.T., Ros, J., Cabiscol, E., Sorribas, A., Herrero, E., 1999. Grx5 glutaredoxin plays a central role in protection against protein oxidative damage in Saccharomyces cerevisiae. Mol Cell Biol 19 (12), 8180-8190.
